# Mitochondrial DNA Methylation and Human Diseases

**DOI:** 10.3390/ijms22094594

**Published:** 2021-04-27

**Authors:** Andrea Stoccoro, Fabio Coppedè

**Affiliations:** Department of Translational Research and of New Surgical and Medical Technologies, University of Pisa, 56126 Pisa, Italy; andrea.stoccoro@unipi.it

**Keywords:** mitoepigenetics, mtDNA methylation, D-loop region, mitochondria impairment, human diseases, environmental factors

## Abstract

Epigenetic modifications of the nuclear genome, including DNA methylation, histone modifications and non-coding RNA post-transcriptional regulation, are increasingly being involved in the pathogenesis of several human diseases. Recent evidence suggests that also epigenetic modifications of the mitochondrial genome could contribute to the etiology of human diseases. In particular, altered methylation and hydroxymethylation levels of mitochondrial DNA (mtDNA) have been found in animal models and in human tissues from patients affected by cancer, obesity, diabetes and cardiovascular and neurodegenerative diseases. Moreover, environmental factors, as well as nuclear DNA genetic variants, have been found to impair mtDNA methylation patterns. Some authors failed to find DNA methylation marks in the mitochondrial genome, suggesting that it is unlikely that this epigenetic modification plays any role in the control of the mitochondrial function. On the other hand, several other studies successfully identified the presence of mtDNA methylation, particularly in the mitochondrial displacement loop (D-loop) region, relating it to changes in both mtDNA gene transcription and mitochondrial replication. Overall, investigations performed until now suggest that methylation and hydroxymethylation marks are present in the mtDNA genome, albeit at lower levels compared to those detectable in nuclear DNA, potentially contributing to the mitochondria impairment underlying several human diseases.

## 1. Introduction

Mitochondria are double-membrane organelles which play a vital role in a variety of key biological functions, including production of ATP through oxidative phosphorylation (OXPHOS), apoptosis via caspase-dependent and independent mechanisms, regulation of calcium homeostasis and production of reactive oxygen species (ROS) [1]. Mitochondria contain their own DNA, a molecule of 16,569 bp inherited in a maternal, non-Mendelian fashion. Each mitochondrion contains multiple copies of mtDNA, which is distinctly different from nuclear DNA in several ways. In part, this can be explained by the endosymbiotic theory, which states that mitochondria evolved from an alpha-proteobacterium that invaded eukaryotic cells [2]. Indeed, similar to the DNA of prokaryotic cells such as bacteria, mtDNA is a circular, double-stranded DNA molecule formed by the heavy (H) and the light (L) strand, lacking histones and organized into tightly packed nucleoprotein complexes called nucleoids [3]. 

The mitochondrial genome comprises 37 genes, 13 of which encode for polypeptides required for the electron transport chain (ETC), including seven genes encoding for subunits within complex I (*MT-ND1*, *MT-ND2*, *MT-ND3*, *MT-ND4*, *MT-ND4L*, *MT-ND5*, *MT-ND6*), one for complex III (*MT-CYB*), three for complex IV (*MT-CO1*, *MT-CO2*, *MT-CO3*) and two for complex V (*MT-ATP6*, *MT-ATP8*), in addition to two ribosomal RNAs (*MT-RNR1* and *MT-RNR2*) and 22 transfer RNAs [4]. Unlike nuclear DNA, which contains at least one promoter per gene, mtDNA contains only three promoter regions, including LSP for genes encoded by the L-strand, and HSP1 and HSP2 for the genes encoded by the H-strand, that transcribe multiple genes at once to produce polycistronic transcripts [5]. These promoters are located within or in the vicinity of a 1124-bp locus, known as the mitochondrial displacement loop (D-loop) region, which also contains the origin of replication of the H-strand. Several proteins encoded in nuclear DNA regulate both mtDNA transcription and replication, including the mitochondrial RNA polymerase (POLRMT), the transcription and mtDNA maintenance factor (TFAM), the transcription specificity factors (TFB1M and TFB2M) and the transcription termination factor (mTERF) [6].

Growing evidence is showing that also epigenetic mechanisms could contribute to the regulation of both mitochondrial DNA transcription and replication, leading to coining the term “mitoepigenetics” [7]. Epigenetics refers to heritable changes in gene regulation that are not due to changes in the DNA sequence, including DNA methylation, histone modifications and non-coding RNA-mediated mechanisms, which are tissue- and cell-specific and may change overtime as a result of aging, disease or environmental stimuli [8]. In the last few years, there has been a growing interest in the potential role of epigenetic mechanisms in the pathogenesis of various human diseases, including cancer, cardiovascular diseases and neurodegeneration. Epigenetic alterations have started being considered as valuable biomarkers for the diagnosis and prognosis of certain types of cancer, as well as for neurological and autoimmune diseases, and, given their reversible nature, it is widely accepted that epigenetic mechanisms provide promising opportunities for interventions aimed to prevent or ameliorate disease symptoms by targeting lifestyles and using epigenetic-based therapy [9]. 

However, compared to the epigenetic regulation of nuclear DNA, the mechanisms of mtDNA epigenetic regulation have been less investigated, and only in the last few years has there been a growing interest in this field [7]. mtDNA is devoid of histones, and its structure is different from that of nuclear chromatin, meaning that one of the most investigated epigenetic modifications occurring in this molecule is DNA methylation [10]. In this review, we discuss the role of mitoepigenetics in the regulation of mitochondrial metabolism, highlighting the role of DNA methylation inside this organelle and its impact on human diseases. We also discuss the possible impact of the nuclear genetic background and of environmental factors in the regulation of mtDNA methylation.

## 2. Epigenetic Mechanisms

Epigenetic mechanisms have been largely investigated in nuclear DNA and include DNA methylation, post-translational modifications on histone tails and nucleosome positioning that tightly regulate gene expression levels, chromatin folding and the three-dimensional structure of the nuclear genome [11,12,13]. DNA methylation is the most widely studied epigenetic mechanism and consists in the addition of a methyl group to cytosine, forming 5-methylcytosine (5-mC) [14]. The reaction is mediated by enzymes called DNA methyltransferases (DNMTs) which use S-adenosylmethionine (SAM), produced during the one-carbon metabolism, as the methyl donor compound [15]. In the nuclear genome, DNA methylation occurs primarily on cytosines followed by a guanine, called CpG sites. Sites of CpG clusters are called CpG islands, and when a CpG island in the promoter region of a gene is methylated, the expression of that gene is usually repressed. By contrast, cytosine methylation in gene bodies could be related to both the active and the repressed transcriptional state depending on the tissue in which it occurs [16]. CpG dinucleotides are also located in repetitive or centromeric sequences, where their methylation is associated with the maintenance of chromosomal stability and with prevention of translocation events [17]. DNA methylation also occurs at non-CpG sites (CpA, CpT and CpC) in the nuclear genome, but non-CpG methylation is restricted to specific cell types, including neurons, glial cells and embryonic stem cells [18]. Hydroxymethylcytosine (5-hmC) is another modification of cytosine mediated by members of the ten-eleven translocation (TET) protein family, which is important for proper gene transcription as it directs the dynamic remodeling and organization of the chromatin structures [19].

Histones are the most abundant proteins associated with nuclear DNA and aggregate each other, forming the histone octamer around which DNA is wrapped, creating the nucleosome. The N-terminal tails of histones may undergo several post-translational modifications, including acetylation, methylation, phosphorylation, ubiquitination and ADP ribosylation. These changes influence the chromatin structure, facilitating or inhibiting gene transcription [11]. For example, acetylation of lysine residues leads to a more relaxed chromatin structure, allowing greater access of transcriptional activators to the underlying genomic sequence [20]. 

In addition to histone modifications and DNA methylation, a further layer of epigenetic regulation of gene expression and chromatin state exists at the level of short (<200 nt) and long (>200 nt) non-protein coding RNAs (ncRNAs) [21]. miRNAs (22–25 nt) are the most studied ncRNAs and regulate gene expression in a sequence-specific manner. In fact, they bind to the 3′ untranslated region of target mRNA molecules and mediate their post-translational regulation, leading to either degradation or translational inhibition, depending on the degree of sequence complementarity [22].

The different structure, nature and complexity of the mitochondrial genome with respect to the nuclear one and the absence of histone proteins have led researchers to question, for several years, about the existence of mitochondrial epigenetic mechanisms [23]. However, mounting evidence is revealing a bidirectional crosstalk between the nuclear and the mitochondrial genome to tightly regulate metabolic reactions and gene expression levels according to the cellular demands, primarily acting through epigenetic mechanisms [15,24,25].

## 3. Mitoepigenetics

The term “mitoepigenetics” has been coined to indicate the epigenetic mechanisms that regulate the mitochondrial genome, but recently, it has more broadly been used to include the complex interactions between mitochondria and epigenetic mechanisms [7,26]. Indeed, mitochondria provide key metabolites to the nucleus, including β-nicotinamide adenine dinucleotide, ATP, α-ketoglutarate and acetyl coenzyme A that are co-substrates required for epigenetic processes [1]. Moreover, mtDNA variants may have effects on the transcription of genes in the nuclear genome through epigenetic mechanisms, as the removal of mtDNA or changes in mtDNA haplogroups have been found to be associated with differences in nuclear DNA methylation levels [27,28,29], suggesting an important epigenetic interplay between the two genomes. Recently, it has also been observed that changes in the mtDNA copy number influence nuclear DNA methylation at specific loci and result in differential expression of specific genes that may impact human health and disease via altered cell signaling [30]. On the other hand, nuclear epigenetics modulates mitochondrial function, as many of the mitochondrial proteins are nuclear-encoded. For example, the *POLGA* nuclear gene, which encodes the enzyme polymerase gamma, a subunit of the polymerase responsible for mtDNA replication and repair, is regulated by epigenetic mechanisms, and its methylation status regulates the mtDNA copy number [31,32]. Overall, it is emerging that epigenetic mechanisms are fundamental for a bidirectional crosstalk between the nuclear and mitochondrial genomes that allows a coordinated gene expression and metabolic response to different cellular conditions and energy demands [15,24,25].

Concerning epigenetic mechanisms occurring within mitochondria, few studies have demonstrated the presence of ncRNAs inside these organelles, both nuclear- and mitochondria-encoded, which seem to be mainly involved in the communication between the nucleus and the mitochondria, at both anterograde and retrograde signaling levels [reviewed in 6]. Moreover, although mtDNA organization is not supported by histones, mtDNA interacts with multiple proteins, including TFAM, mitochondrial single-stranded DNA binding protein and Twinkle mtDNA helicase to create nucleoprotein structures called nucleoids, which could be regulated by epigenetic mechanisms, such as acetylation and phosphorylation [33]. However, many more studies have focused on the role of mtDNA methylation and hydroxymethylation as mitoepigenetic mechanisms, and these epigenetic marks will be the main focus of the present review. 

Although some authors failed to find DNA methylation in the mitochondrial genome [34,35,36], the presence of methylation in mtDNA, as well as the identification of a DNA methyltransferase activity in mitochondria, has been observed for the first time in studies performed more than forty years ago in loach embryos, and mouse and hamster samples [37,38,39]. Subsequent studies showed the presence of mtDNA methylation in human and mouse fibroblasts [40,41], but not in gastric and colorectal cancer human tissues [42]. Investigations by Rebelo and co-workers [43] and Shock and co-workers [44] rekindled the interest in mitoepigenetics, since they not only identified the presence of mtDNA methylation marks in human and mouse cell cultures using more modern techniques than those used in previous studies but also suggested a functional role of these marks in mitochondrial metabolism. Indeed, Rebelo et al. [43] showed that DNA methylation associated with the accessibility of TFAM to mtDNA, thus regulating mtDNA replication, whilst Shock and collaborators showed that mtDNA methylation induced by the binding of the DNMT1 enzyme to the D-loop control region was able to regulate the expression of *MT-ND6* and *MT-ND1* genes [44]. Several researchers have subsequently corroborated these observations. For example, by using biophysical approaches, it has been recently demonstrated that CpG methylation in the D-loop region regulates TFAM-dependent activities in vitro, strictly regulating mtDNA gene transcription [45]. Moreover, while the presence of DNMT1 in mitochondria has been further confirmed by other research groups [46,47], also the presence of the de novo DNMT3A and DNMT3B [48,49,50] and of the TET enzymes [51,52] has been identified in mitochondria. Several authors also observed a significant correlation between the mtDNA methylation levels and mitochondrial copy number, as well as between mtDNA methylation and mtDNA gene expression, thus further suggesting the important role of DNA methylation in mitochondrial regulation [53,54,55,56,57,58,59].

These studies highlighted that mtDNA could be subjected to DNA methylation and hydroxymethylation by the same enzymes of the nuclear genome. However, some differences between mtDNA and nuclear DNA methylation exist. Indeed, contrarily to genomic DNA, mtDNA showed to possess high levels of non-CpG versus CpG methylation, which is not symmetrical on both strands, being biased toward the L-strand, and it seems that depending on the cell type and context of cytosine methylation, mtDNA methylation can play a role in the regulation of mtDNA gene expression or mtDNA replication [46,50,54,60,61,62]. Additionally, 5-hmC has been detected in mtDNA, and there is evidence that 5-hmC marks are dynamic in nature and are enriched in the upstream of gene start site regions and in the gene body, similarly to nuclear genes [63]. Moreover, recent studies reported that human mtDNA is particularly enriched in N^6^-methyldeoxyadenosine (6-mA), which is usually widespread in prokaryotes but less present in mammals’ nuclear genome, an epigenetic mark which affects mitochondrial DNA transcription, replication and activity [64,65,66]. These studies clearly suggest that there is a specific regulation of mtDNA methylation, and it is of outmost importance to know how these regulatory mechanisms work in order to better understand how mitochondrial function is regulated.

## 4. Altered mtDNA Methylation and Human Diseases

The role of altered nuclear epigenetic mechanisms is now well established in the etiology of various human diseases [9]. On the other hand, information regarding a potential role of mitoepigenetic mechanisms in human diseases is only recently emerging [10,23,67]. This discrepancy is mainly due to the limited availability of methodologies for studying mtDNA methylation and hydroxymethylation until a few years ago. However, the application of different approaches, including 5-mC immunoprecipitation, bisulfite sequencing, pyrosequencing, next-generation sequencing, liquid chromatography tandem mass spectrometry and ELISA assays [68], has largely increased the possibility of studying mtDNA modifications in recent years, and several authors focused their investigations on the search for mitoepigenetic alterations in various human diseases, and particularly on differentially methylated and hydroxymethylated mtDNA regions.

### 4.1. Evidence of Altered mtDNA Methylation in Cancer

The association between mtDNA methylation and cancer has been largely investigated (Table 1).

Although some authors did not identify the presence of mtDNA methylation in cancer cell lines, in malignant and non-malignant tissue from patients with gastric and colorectal cancer, in adenomas and in cervix cancer [42,69,72], findings of other authors suggested an involvement of mtDNA methylation impairment in tumorigenesis. Feng and co-workers analyzed D-loop methylation in 44 colorectal cancer tissues and in their corresponding adjacent non-cancerous tissues, finding that the D-loop of non-cancerous tissue was methylated, while in the majority of cancer tissues, the D-loop was unmethylated [58]. Moreover, ND2 protein expression was higher in cancer tissues compared to adjacent normal tissues, suggesting that D-loop methylation levels may play a role in regulating *MT-ND2* expression. Gao and co-workers correlated methylation of the D-loop region with the mtDNA copy number and ND2 protein expression in 65 colorectal cancer specimens and their corresponding non-cancerous tissues [55]. The methylation rate of the D-loop region in all the 65 colorectal cancer tissues was markedly reduced when compared with that of their corresponding non-cancerous tissues. In addition, the mtDNA copy number and ND2 protein expression were increased in colorectal cancer tissues with respect to non-cancerous ones. Demethylation of the D-loop region was associated with an elevated mtDNA copy number and an increased ND2 expression. Furthermore, the mtDNA copy number and ND2 expression in Caco-2 cancer cells were significantly increased after treatment with the demethylating agent 5-aza-2′-deoxycytidine. Similarly, Lovo and Colo-205 colorectal cancer cell lines treated with 5-aza-2′-deoxycytidine showed lower D-loop methylation levels along with a higher content of the mtDNA copy number compared to non-treated cells [70]. Altered mtDNA methylation has also been associated with breast cancer. Indeed, in a study performed in the peripheral blood of female individuals belonging to five families with one breast cancer patient, aberrant mtDNA methylation of the D-loop region was correlated with breast cancer risk [71]. Interestingly, the authors observed that the D-loop region demonstrated a familial-specific mtDNA methylation pattern among the five families and suggested that D-loop methylation was maternally inherited [71]. In a following study, a whole-mtDNA genome methylation analysis was performed in human model cells of breast cancer and hepatocarcinoma, as well as in the corresponding normal cells, finding that the methylation pattern was markedly different between normal and cancer cells primarily in a non-CpG context [61]. By using cellular models of glioblastoma and osteosarcoma, it was found that mtDNA methylation levels tended to decrease during tumor progression, potentially contributing to the increase in mtDNA [73]. These changes also correlated with transcriptional changes of *MT-ND5* and *MT-ND6* genes during tumorigenesis. Interestingly, after tumors had restored sufficient mtDNA to initiate tumorigenesis, higher levels of 5-mC over the D-loop were acquired and the authors suggested that this could occur to potentially restrict further replication of mtDNA [73]. Recently, whole-mtDNA genome methylation has been investigated by means of nanopore sequencing in oral squamous cell carcinoma cell cultures with different sensitivity to cisplatin, observing that enhanced cisplatin resistance was not influenced by the methylation status of the mitochondrial genome [74]. However, the authors suggested that mtDNA methylation in the gene bodies promoted the expression of the genes, presumably by affecting the post-transcriptional modifications of polycistronic mitochondrial mRNAs, since in one cell culture, they found hypermethylation of *MT-CO1* and *MT-CYB* genes with concomitant high expression levels [74]. By using the same technique, increased CpG and CpH mtDNA methylation levels have been observed in head and neck tumor samples when compared to their matched adjacent normal tissues [75].

### 4.2. Altered mtDNA Methylation in Metabolic and Cardiovascular Diseases

Metabolic syndrome represents a cluster of clinical conditions, including high blood pressure, insulin resistance, lipid abnormalities and obesity that are associated with increased risk of diabetes and cardiovascular diseases (CVDs) [76]. Among the pathophysiological abnormalities underlying the development of metabolic syndrome, impaired mitochondrial oxidative phosphorylation and mitochondrial biogenesis seem to play a pivotal role, although the molecular mechanisms underlying the mitochondrial impairment remains largely unexplored [77]. Studies performed in the last few years are suggesting that mitochondrial impairment that characterizes obesity, insulin resistance, diabetes and CVDs could be derived from alterations in mtDNA methylation (Table 2).

Indeed, insulin resistance has been associated with a reduction in the mtDNA copy number due to increased D-loop methylation levels in the leukocytes from obese individuals [80]. Moreover, D-loop methylation levels have been linked to increased diabetes risk and have been proposed as indicators of early-stage prediabetes [81]. Furthermore, treatment of retinal endothelial cells with high levels of glucose increased D-loop methylation levels, and the retinal microvasculature from human donors with diabetic retinopathy presented a similar increase in D-loop methylation and a decrease in mtDNA transcription [79]. Increased D-loop methylation levels were also observed in buccal swab DNA collected from overweight female subjects, when compared to lean female subjects [82]. Moreover, a specific CpG site in the D-loop region associated with impaired body composition, evaluated by body mass index, waist to height ratio and bioimpedance measurements. Interestingly, body composition impairment was well predicted by a combined variable including the mtDNA copy number and D-loop methylation [82]. Increased D-loop methylation levels were also detected in retinal microvasculature samples from a type 2 diabetic rat model when compared to type 1 diabetic rats or high-fat diet rats [84].

The first evidence of a potential involvement of an impairment of mtDNA methylation in the etiology of CVDs was obtained by a study performed in platelet mitochondria of 10 CVD patients and 17 healthy individuals, in which higher methylation of *MT-CO1*, *MT-CO2*, *MT-CO3* and *MT-TL1* genes in CVD patients was observed [78]. In a following study performed in human samples, in cell cultures and in mouse models of arterial stenotic/occlusive disease, it was observed that in vascular smooth cells, the enzyme DNMT1 translocates to the mitochondria in response to pro-proliferative stimuli and induced D-loop hypermethylation [85]. The D-loop hypermethylation led to repression of mtDNA transcription, inducing mitochondrial dysfunction and reduction in ATP production, thus impairing vascular smooth cell contractility in the context of vascular restenosis or occlusion [85]. Recently, in an attempt to identify mitochondria activity differences between stable coronary artery disease (SCAD) and acute coronary syndrome (ACS), the mtDNA copy number and methylation levels of the nuclear peroxisome proliferator-activated receptor gamma coactivator 1-alpha (*PPARGC1A*) and of the D-loop region were evaluated in peripheral blood leukocytes of 50 patients with SCAD and of an equal number of individuals with ACS [86]. The authors observed that SCAD patients had a higher content of the mtDNA copy number as well as lower D-loop methylation levels, thus suggesting that an altered mtDNA copy number and mtDNA methylation may affect the clinical phenotype of coronary artery disease [86].

To better understand why some individuals with obesity develop CVDs while others remain disease-free, methylation levels of several mtDNA genes, including *MT-CO1*, *MT-CO2*, *MT-CO3*, *MT-TL1* and *MT-TF*, and of the D-loop and light-strand origin of replication (MT-OLR) regions were evaluated in platelet mtDNA from 200 adults with overweight and obesity, of whom 84 developed cardiovascular disease [83]. The authors found that methylation levels of *MT-CO1*, *MT-CO3* and *MT-TL1* were higher in subjects that developed CVDs, suggesting that methylation levels of these genes could be strong predictors of future CVDs incidence in adults with overweight and obesity [83].

### 4.3. Modulation of mtDNA Methylation in Aging and Senescence

The most common causes of the majority of diseases are senescence and aging, likely due to the accumulation of tissue and organ dysfunctions over time [87]. Decline in mitochondrial quality and activity has been associated with normal aging, but also with the development of a wide range of age-related diseases, and it is now well accepted that mitochondria contribute to specific aspects of the aging process including cellular senescence and age-dependent decline in cellular activity [88]. To better elucidate the molecular alteration underlying mitochondria involvement in cell senescence and aging, some authors investigated a potential role of mtDNA methylation (Table 3). 

In order to explore a possible role of mtDNA methylation in relation to replicative senescence, D-loop region methylation was investigated in replicative as well as in senescent human and mouse endothelial cells [53]. Demethylation of the D-loop region with an increased mtDNA copy number was observed in senescent cells compared to proliferative endothelial cells [53]. Using senescent mesenchymal stem cells, three CpG sites located in various mitochondrial genes were found to be hypomethylated in senescent cells as compared to non-senescent ones. Of particular interest was the finding that methylation of one of these CpG sites, located in the *COX1* gene, led to repression of *COX1* gene expression in parallel with the onset of senescence [57]. More recently, also increased methylation levels of the *COX2* gene, accompanied by decreased protein expression, have been associated with cell senescence [92]. Interestingly, cell treatment with 5-aza-2′-deoxycytidine inhibited *COX2* methylation and downregulated *COX2* expression, promoting cell proliferation and delaying cell aging [92].

While no presence of 5-mC in mtDNA has been detected during mouse oocyte maturation, aging and early embryo development [91], aging in adult individuals has been correlated with changes in mtDNA methylation and hydroxymethylation. A study performed in mouse brain samples found that levels of DNA hydroxymethylation decreased in the frontal cortex, but not in the cerebellum, during aging [52]. In a study performed in peripheral blood of 381 individuals ranging from 38 to 107 years of age, methylation levels of the *MT-RNR1* gene were positively associated with increasing age [89]. Interestingly, subjects with higher methylation levels also exhibited a higher mortality risk than those with lower methylation levels, thus suggesting that the methylation of the analyzed CpG sites may reflect a condition of the cell or of the organism to survive. A later study investigated methylation levels of 133 CpG sites of mtDNA in the peripheral blood of 82 female individuals aged 18–91 years, finding detectable methylation in 54 CpG sites, two of which located within the *MT-RNR1* gene showed a strong inverse correlation with subject age [90]. 

### 4.4. Altered mtDNA Methylation in Neurodegenerative Diseases

Multiple lines of evidence suggest that mitochondrial dysfunction is involved in the pathogenesis of neurodegenerative diseases, especially in Alzheimer’s disease (AD), Parkinson’s disease (PD) and amyotrophic lateral sclerosis (ALS), leading some researchers to investigate the potential involvement of mitoepigenetic mechanisms in the etiology of these diseases (Table 4).

Early in 2011, Chestnut and co-workers investigated the global 5-mC content and the DNMT protein levels in nuclei and mitochondria from both brain and spinal cord motor neurons of mice, as well as in cortical motor neurons of 12 ALS patients [48]. The authors revealed that motor neurons engaged epigenetic mechanisms to drive apoptosis, involving up-regulation of DNMTs that increased global DNA methylation in both nuclei and mitochondria [48]. Subsequently, another study from the same research group revealed that mtDNA methylation patterns and mitochondrial DNMT3A levels were abnormal in the skeletal muscles and spinal cord of pre-symptomatic ALS mice carrying mutations in the human superoxide dismutase 1 gene (*SOD1*), which included DNMT3A up-regulation, increased *MT-RNR2* gene methylation and decreased D-loop region methylation [49]. Altered D-loop methylation levels have also been observed in the peripheral blood of sporadic and *SOD1* ALS patients when compared to both ALS patients with mutations in *FUS*, *TARDBP* and *C9orf72* and to control subjects who are noncarriers of ALS-linked gene mutations [96,100]. 

Regarding Alzheimer’s disease, a non-significant increase in 5-hmC levels was observed in post-mortem mtDNA brain samples of seven late-onset AD patients with respect to five control subjects [93]. A later study revealed increased methylation levels of the mtDNA D-loop region and reduced *MT*-*ND1* methylation levels in the enthorinal cortex of eight patients with AD-related pathology with respect to healthy control brains [94]. Interestingly, the degree of D-loop region methylation was higher in patients in the early disease stages than in later stages, and these results were corroborated by a dynamic pattern of methylation of this region that was observed in an AD mouse model along with the progression of the disease [94]. We evaluated D-loop methylation levels in the peripheral blood of 133 late-onset AD patients and 130 controls, observing a significant 25% reduction in DNA methylation levels in the patient group [95]. A decrease in D-loop methylation levels and an increase in *mt-Rnr1*, *Cytb* and *Cox-2* gene methylation with a concomitant reduction in both the mtDNA copy number and the mitochondrial gene expression have been observed in the hippocampus of an AD mouse model when compared to wild-type mice [97,101]. An interesting paper reported that treatment of a blood barrier cell line (hCMEC/D3) with Aβ peptide induced global mtDNA hypermethylation, and that this mtDNA methylation status persisted after the removal of Aβ, inducing a cerebrovascular endothelial damage memory that likely contributes to AD progression [98]. 

Few studies investigated a potential involvement of altered mitoepigenetic mechanisms in Parkinson’s disease. Decreased D-loop methylation levels were observed in the substantia nigra of 10 PD patients with respect to healthy matched controls [94]. More recently, no differences in platelet mtDNA methylation levels at *MT-TL1* and *MT-CO1* genes were observed between PD patients and control subjects [99].

Taken together, these studies suggest that mitochondria impairment characterizing neurodegenerative diseases could be related to altered mitoepigenetic mechanisms, with the potential to provide new insights into their etiopathogenesis and new biomarkers of diagnosis and progression of these diseases.

### 4.5. Altered mtDNA Methylation in Other Diseases

MtDNA methylation alterations have also been associated with other human diseases than cancer, cardiovascular diseases and neurodegeneration (Table 5). 

Infantino and co-workers observed decreased SAM availability in Down’s syndrome lymphoblastoid cells, with a consequent reduction in mitochondria methyl uptake, leading to mtDNA hypomethylation [102]. Analysis of mtDNA methylation in liver biopsies from patients with non-alcoholic steatohepatitis revealed that *MT-ND6* methylation was significantly higher than that observed in individuals with simple steatosis [103]. Moreover, *MT-ND6* mRNA expression was decreased in NASH patients. Interestingly, the methylation status of the *MT-ND6* gene was inversely correlated with physical activity, suggesting that epigenetic changes in mtDNA are potentially reversible [103]. Methylation analysis in DNA extracted from fetal cord blood of newborns from mothers with placenta insufficiency showed that D-loop methylation levels were decreased in the cases with respect to controls and associated with poorer fetal outcomes, as indicated by their correlation with gestational age, fetal weight and umbilical vein oxygen partial pressure [104]. No alterations in mtDNA methylation were detected in individuals with major depressive disorder and attention-deficit hyperactivity disorder when compared to healthy control subjects [105,106].

## 5. Effects of Environmental Exposure and of Nuclear Genetic Variants on mtDNA Methylation

It is well known that the individual genetic background and environmental factors can impact the health status by modulating nuclear epigenetic mechanisms [107]. Recent research clearly suggests that also mtDNA methylation and hydroxymethylation patterns can be modulated by exposure to various environmental agents and by nuclear genetic variants (Table 6). 

It has been reported that mtDNA methylation is modulated by exposure to particulate matter (PM_1_ and PM_2.5_), air benzene, traffic-derived elemental carbon [56,108,113], particle-containing welding fumes [120], endocrine disruptors [109], maternal smoking [112,118,129], chrome [117], arsenic [59] and iron [121], as well as to the pharmacological agent valproic acid [51], to the demethylating agents 5-azacytidine and vitamin C [70,122] and to HIV infection and cocaine [125]. Variations in mtDNA methylation patterns have also been associated with various endogenous metabolites, including thyroid hormones [119], homocysteine [114,126], betaine [110], glucose [79,80], l-carnitine [124], lipopolysaccharides [127] and mitochondrial-derived peptides [123]. Moreover, nutrients from the diet, including lipids and fructose, were also able to modulate mtDNA methylation levels in animal models [111,115,116]. 

The main mitochondrial region analyzed in those studies was the D-loop region, which has been found to be negatively associated with PM_2.5_ blood levels [113], welding fumes [120], arsenic [59], maternal smoking during pregnancy in placenta and foreskin [112], 5-azacytidine [70] and cord blood thyroid hormones levels in placenta [119]. On the other hand, increased D-loop region methylation has been associated with PM_2.5_ levels together with maternal smoking in placenta [56,129], hyperhomocysteinemia [114], high glucose levels [79], high lipid levels [115], l-carnitine [124] and mitochondrial-derived peptides [123]. Another mtDNA region frequently investigated is the *MT-RNR1* gene whose methylation levels have been found to be positively associated with PM_1_ [108], PM_2.5_ [56] and maternal smoking [118] and negatively associated with chrome exposure [117], thyroid hormones [119] and olive oil consumption [111]. However, many other mtDNA regions have been found to be sensitive to environmental cues, thus suggesting that mtDNA could be used as a sensor of environmental stressors.

There is also evidence that some nuclear genetic variants could impact mtDNA methylation. For example, a strong influence from the nuclear genome was observed on mtDNA methylation patterns in glioblastoma and osteosarcoma cells, since the same mtDNA genotype under different nuclear genomes associated with differential mtDNA methylation patterns [73]. Indeed, by using cell cultures with the same mitochondrial genome but with different nuclear genomes, derived from osteosarcoma or glioblastoma cells, several differentially methylated CpG sites located across the whole genome have been detected [73]. More recently, polymorphisms of genes involved in one-carbon metabolism, namely, *MTRR* 66A > G and *DNMT3A* −448A > G, have been found to be significantly associated with D-loop methylation levels [128]. The importance of one-carbon metabolism in mitochondria metabolism has also been shown by a study performed in individuals belonging to three families with segregate mutations in the *SLC25A26* gene, which encodes for the mitochondrial S-adenosylmethionine (SAM) transporter, required for SAM uptake from the cytosol [130]. Probands of these families showed high clinical heterogeneity, which ranged from neonatal mortality resulting from respiratory insufficiency and hydrops to childhood acute episodes of cardiopulmonary failure and slowly progressive muscle weakness. The authors also showed that *SLC25A26* mutations caused severe abrogation of SAM transport capacity, leading to mitochondrial methylation insufficiency, which led to altered mitochondria RNA stability and altered protein modifications [130]. Additionally, lymphoblastoid cells of individuals with Down’ syndrome, a genetic disorder caused by the presence of all or part of a third copy of chromosome 21, have been found to be depleted in mitochondrial levels of SAM, with concomitant global mtDNA hypomethylation [102]. The authors suggested that this could be due to the overexpression of cystathionine-beta-synthase, located on chromosome 21, which modifies the levels of several intermediates of the cellular one-carbon metabolism in Down’s syndrome, including SAM. Interestingly, two rare hereditary neurodegenerative diseases, the autosomal dominant cerebellar ataxia, deafness and narcolepsy (ADCA-DN) and the hereditary sensory neuropathy with dementia and hearing loss (HSN1E), whose phenotypes share some clinical features typical of mitochondrial diseases, including mitochondrial dysfunction, optic atrophy, peripheral neuropathy and deafness, have been linked to dominant pathogenic mutations in the *DNMT1* gene [131]. 

Methylation levels of mtDNA have also been found to be sensitive to mutations in genes related to ALS pathogenesis [96,100]. Indeed, in individuals belonging to families with segregate ALS-linked mutations, we observed that individual carriers of *SOD1* and *C9orf72* mutations had significantly higher levels of the mtDNA copy number than noncarriers of ALS-linked mutations, but only *SOD1* mutation carriers showed a significant reduction in D-loop methylation levels [96]. On the other hand, *TARDBP* and *FUS* carriers showed no significant increase in the mtDNA copy number and no significant decrease in D-loop methylation compared to noncarriers of ALS-linked mutations. In a later study, we observed that both *SOD1*-mutant and sporadic ALS patients had lower D-loop methylation levels compared to controls, while *C9orf72* ALS patients showed similar D-loop methylation levels to control subjects [100].

## 6. Debate on the Existence and Function of mtDNA Methylation

Although the presence of different DNMTs and epigenetic marks, including 5-mC and 5-hmC, inside mitochondria has been reported by several authors, the presence and biological function of mtDNA methylation have been hotly debated in recent years. Indeed, some researchers reported that mtDNA methylation is a very rare event, or even absent, suggesting that the literature reporting the presence of methylation marks in mtDNA could be biased by technical limitations [34,35,36,132]. Overall, these studies suggest that, although the possibility of the presence of low levels of mtDNA cannot be ruled out, it is doubtful that a biologically significant function should be considered at the extremely low frequency of CpG methylation detected.

Evidence of the absence of DNA methylation marks in mitochondria was reported about forty years ago [133,134]. However, the first study conducted in more recent years reporting the absence of methylation in mtDNA and performed using modern techniques, including bisulfite sequencing and next-generation sequencing, was conducted in 2013 in a human colon cancer cell line (HCT116) and in primary human cells [34]. The authors found a lack of an appreciable amount of methylated cytosines, arguing that it is highly unlikely that CpG methylation plays any role in the direct control of the mitochondrial function, confirming this evidence by analyzing ten genome-wide bisulfite sequencing published datasets. Of note, van der Wijst and collaborators investigating mtDNA methylation in different human cell lines, including HCT116 cells, found evidence of methylation levels, although low, in all the cells investigated except the HCT116 cell lines in which no evidence of 5-mC was detected [54]. Moreover, by reanalyzing the datasets used by [34], a recent study confirmed that CpG methylation was low, but when the analysis was carried out in a strand-specific manner, high non-CpG methylation was observed in the L-strand [50], meaning that the existence of mtDNA methylation cannot be ruled out in these datasets. In later studies, it was suggested that the circular structure of mtDNA could interfere with bisulfite conversion, which is a preliminary sample preparation step for several DNA methylation techniques, thus leading to overestimation of mtDNA methylation. During DNA treatment with bisulfite, unmethylated cytosines are converted to uracil residues, while methylated cytosines remain unchanged; the subsequent PCR amplification or extension step introduces thymine to the positions of uracil and cytosine to those of 5-mC. Liu and co-workers showed that that the methylation values obtained from linear mtDNA were significantly lower than those obtained from circular mtDNA and reported that CpG methylation in human mtDNA is a very rare event at most DNA regions and that it seems that such low level of mtDNA methylation would have limited or absent functional significance in the control of mitochondrial gene expression [132]. However, the authors also reported that average methylation levels of the 83 CpG sites investigated were less than 2% with the exception of two CpG sites within the D-loop region, which showed 5% of methylation, concluding that the higher methylation status at this region might play a role in the control of mtDNA replication [132]. Additionally, other studies have shown that the circular structure of mtDNA can influence bisulfite treatment and the detection of mtDNA methylation [35,135]. In a later study, the mtDNA methylation status was investigated in mouse embryonic stem cells and liver and brain tissues by means of three distinct methods for detecting 5-mC, namely, bisulfite sequencing, McrBC (an approach that uses enzymes sensitive to CpG methylation) and liquid chromatography mass spectrometry (LC/MS), finding that methylated cytosines are fairly low, thus arguing that it is unlikely that mtDNA methylation plays a role in mtDNA gene expression or mitochondrial metabolism [36]. It should be noted that by means of LC/MS, which is more sensitive than the other two methods in detecting 5-mC, weak methylation signals, corresponding to 18–30 5-mC residues per molecule of mtDNA, were detected [36]. Collectively, these studies suggest that it is doubtful that a biologically significant function should be considered at the extremely low CpG methylation levels detected in mtDNA. Moreover, they highlight that methodological precautions should be adopted when studying mtDNA methylation, as the mtDNA circular structure, the procedures used to extract DNA samples, the DNA bisulfite treatment, primer design and the use of inadequate control templates could lead to erroneous methylation and hydroxymethylation quantification [132,136]. 

However, several other studies that have carefully followed the necessary precautions for the study of mtDNA methylation, or that used techniques for which bisulfite treatment was not necessary, successfully identified the presence of methylation in the mitochondrial genome [61,73,84,110,127,137]. For example, Patil and co-workers performed a whole-genome bisulfite sequencing after linearization of mtDNA, finding a higher degree and frequency of methylation, particularly in the L-strand when compared to the H-strand in various human cell cultures [61]. Similarly, after linearizing mtDNA and using mtDNA methylation-negative and positive controls for bisulfite sequencing, an average CpG methylation of 10% was detected over the mtDNA genome of tumor cell cultures [73]. To overcome the possibility that the mtDNA circular structure might also affect immunoprecipitation-based methods that do not require bisulfite treatment, some authors performed a denaturation step before the incubation of mtDNA with 5-mC or 5-hmC antibodies, finding, however, the presence of methylated and hydroxymethylated cytosines across the D-loop region [73,110,122]. Moreover, a study investigating mtDNA methylation in human cells by means of a third-generation sequencing platform, namely, the nanopore sequencer (MinION, Oxford Nanopore), which is a high-throughput nanopore-based single-molecule device that can directly sequence the entire mtDNA molecule, minimizing potential nucleotide errors introduced by PCR amplification, and preserving epigenetic modifications by avoiding the use of bisulfite treatment, revealed clearly detectable levels of mtDNA methylation [74]. Likewise, by using high-coverage nanopore sequencing at the single-molecule level, mtDNA methylation was recently investigated in different sample types and biological conditions [75]. Methylation was overall higher in tissues compared to cell lines, and despite that mtDNA methylation levels were generally low, global and single-base differences were found between cancer tissues and the adjacent healthy tissues. Moreover, although low, the methylation levels detected in mtDNA have been frequently related to changes in both mtDNA gene transcription and mitochondrial replication (see Table 1, Table 2, Table 3, Table 4, Table 5 and Table 6). Furthermore, the application of recent technologies able to overcome the limits for the detection of mtDNA methylation revealed that knockdown of DNMTs perturbs mtDNA methylation and gene expression levels, as well as the mtDNA copy number and oxygen respiration [50,61].

**Figure 1:**
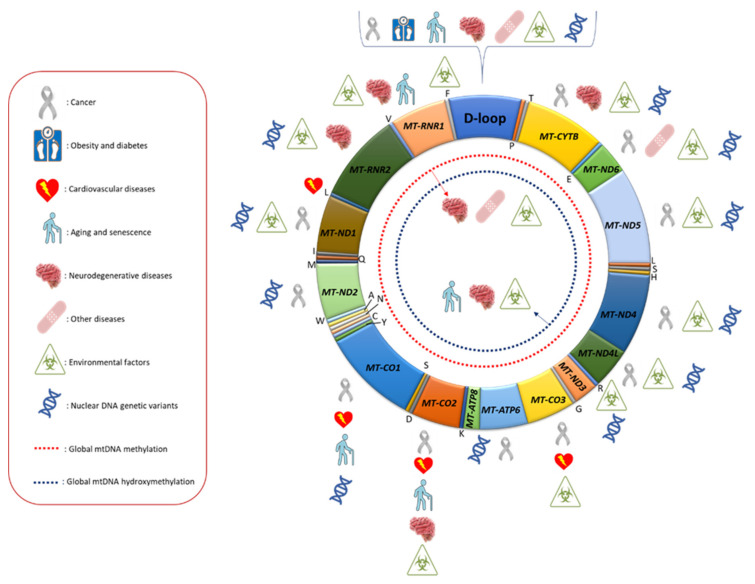
Mitochondrial DNA regions whose methylation levels were found to be associated with different human diseases, environmental factors and nuclear DNA genetic variants. In the red box, the meaning of the symbols used is reported.

Overall, mitoepigenetic investigations performed until now suggest that methylation and hydroxymethylation marks are present in the mtDNA genome, albeit at lower levels compared to those detectable in nuclear DNA, and are altered in different human diseases and sensitive to environmental factors and nuclear DNA genetic variants (Figure 1). Although there are several indications that mtDNA epigenetic modifications could play a role in the biological function of mitochondria, further studies are needed to better elucidate to which extent mtDNA gene expression and replication are regulated by those modifications. In this regard, it should be outlined that from an epigenetic point of view, the mitochondrial genome is largely different compared to the nuclear one, as it lacks nucleosomal chromatin and CpG islands, meaning that the well-known epigenetic regulation of the nuclear genome could be different in mitochondria [138]. A better understanding of the interplay between mtDNA modifications and nucleoid post-transcriptional modifications is pivotal to fully appreciate the biological role of mitoepigenetic mechanisms in the regulation of mitochondrial metabolism. For example, Rebelo and co-workers reported that unbalanced levels, either low or high, of TFAM result in decreasing mtDNA methylation, and this scenario could explain why mtDNA methylation has been observed in some biological models but not in others [43]. Furthermore, it may also be possible that other as yet unidentified epigenetic mechanisms may regulate mtDNA replication and gene expression.

## 7. Conclusions

Alterations in mtDNA gene expression and replication underlie several human pathologies, and a better understanding of the regulation of these mechanisms is desirable to understand the pathophysiology of associated disorders. The discovery of mitoepigenetic mechanisms has opened a new window of research that could provide new knowledge on the mitochondria regulation in both physiological and pathological conditions, with the potential to yield specific biomarkers for several human diseases.

Although the literature reported in the current review indicates that mitoepigenetics has been poorly investigated compared to nuclear epigenetics in human diseases, it is clear that researchers are increasingly interested in this research field. One of the reasons that have delayed mitoepigenetic investigations compared to nuclear epigenetic ones has been the limitation in methodologies until a few years ago [68]. For example, one of the main techniques used in the study of nuclear DNA methylation, the Illumina methylation array, does not cover the mtDNA sequence, so information regarding mtDNA methylation patterns in different human tissues and diseases is still largely missing.

Although some reports suggested the absence of mtDNA methylation in human and mouse mitochondria [34,35,36,132], several other studies showed the presence of consistent levels of mtDNA methylation, and, in particular, the regulatory D-loop region seems to represent one of the mtDNA loci that most frequently undergo methylation and hydroxymethylation, and its methylation levels have been associated with several diseases, including colorectal and breast cancer [55,58,71], obesity [80], insulin sensitivity [81], diabetes [84], arterial stenotic/occlusive disease [85], AD [94,95,97,101], ALS [49,96,100] and PD [94]. Moreover, some studies also showed that D-loop methylation is sensitive to several environmental factors, as well as to nuclear DNA genetic variants (Table 6). 

In conclusion, although our knowledge on the involvement of the mitoepigenetic mechanisms in human diseases is still in its infancy, data obtained until now encourage further addressing this research field, which could shed new light on the pathogenic mechanisms underlying human diseases, potentially providing new biomarkers of disease diagnosis and progression as well as new molecular targets for therapeutic interventions.

## Figures and Tables

**Table 1:** mtDNA methylation studies in cancer.

Experimental Model	Method	mtDNA Region Investigated	Observation	Reference
Cancer cell lines and tissue specimens from patients with gastric and colorectal cancer	Bisulfite-PCR–single-stranded DNA conformation polymorphism (SSCP)	*MT-RNR2*, *MT-CO1* and *MT-CO2* genes	Only unmethylated bands for all analyzed samples were detected	[42]
Human samples of exfoliated cervical lavage positive for HPV16	EpiTYPER and pyrosequencing	Hypervariable segment region	mtDNA methylation was generally low, although few sites showed differences in CpG methylation by disease state	[69]
Colorectal cancer tissues and paired adjacent non-cancerous tissues	Methylation-specific PCR	D-loop region	Hypomethylation of D-loop and increased MT-ND2 protein expression in cancer tissues	[58]
Colorectal cancer tissues and paired adjacent non-cancerous tissues	Methylation-specific PCR	D-loop region	Hypomethylation of D-loop and increased MT-ND2 protein and mtDNA copy number expression in cancer tissues	[55]
Five colorectal cancer cell lines treated with the DNA demethylating agent 5-azacytidine	Sequenom MassARRAY	D-loop region	5-azacytidine induced D-loop demethylation and increased mtDNA copy number content	[70]
Peripheral blood from fifteen female individuals of five families with one breast cancer patient	Bisulfite sequencing	Whole-mtDNA genome methylation	Eight aberrant D-loop methylation sites were correlated with breast cancer. Evidence that mtDNA methylation pattern was maternally inherited.	[71]
Adenoma and normal mucosa paired samples	Whole-genome bisulfite sequencing (WGBS)	Whole-mtDNA genome methylation	Methylation in mtDNA was low in both normal and adenoma tissue and was not associated with mitochondrial gene transcription	[72]
Cellular models of glioblastoma and osteosarcoma	Whole-mtDNA bisulfite sequencing, pyrosequencing and 5-mC and 5-hmC immunoprecipitation	Whole-mtDNA genome methylation	The mtDNA methylation levels decreased during tumor progression along with increased mtDNA copy number. D-loop methylation negatively correlated with MT-*ND5* and *MT*-*ND6* transcription during the tumorigenesis of osteosarcoma cells.	[73]
Normal breast epithelial cells (MCF10A), human breast cancer cells (MCF7), primary human liver (hepatocytes), human hepatocarcinoma cells (HepG2) and primary derived human breast cancer cells (HMEC)	Bisulfite sequencing	Whole-mtDNA genome methylation	Globally, the L-strand displayed a higher degree and frequency of methylation compared to the H-strand. The highest frequency of methylation was detected within a CpT or CpC dinucleotide context, whereas methylation of CpG sites was the least frequently methylated dinucleotide. Methylation patterns display notable differences between normal and cancer cells.	[61]
Cisplatin sensitivity in oral squamous cell carcinoma (OSCC) cell lines, namely, SAS and H103, and stem cell-like tumor spheres derived from SAS	Nanopore sequencing (MinION)	Whole-mtDNA genome methylation	SAS tumor spheres and H103 cells were less sensitive to cisplatin than SAS. Cells with lower mtDNA content were less responsive to cisplatin. Enhanced cisplatin resistance in stem cell-like tumor spheres was not influenced by methylation status of the mitochondrial genome. Hypermethylated of *MT-CO1* and *MT-CYB* in H103 with concomitant higher expression levels of most of the mitochondrial genes, including *MT-CO1* and *MT-CYB*.	[74]
Matched tumor/normal samples of liver (n = 10 pairs) and head and neck (n = 6 pairs) cancers	Nanopore sequencing (MinION and PromethION systems)	Whole-mtDNA genome methylation	Differentially methylated sites between tumor and their matched adjacent tissues detected in head and neck, but not in liver	[75]

**Table 2:** mtDNA methylation studies in metabolic disorders and cardiovascular diseases.

Experimental Model	Method	mtDNA Region Investigated	Observation	Reference
Platelet of 10 patients with cardiovascular disease (CVD) and 17 controls	Pyrosequencing	*MT-CO1*, *MT-CO2*, *MT-CO3*, *MT-TL1*, *MT-ATP6 MT-ATP8* and *MT-MD5* genes	Higher DNA methylation level in CVD individuals than in healthy controls in *MT-CO1*, *MT-CO2*, *MT-CO3* and *MT-TL1* genes	[78]
Bovine retinal endothelial cells and retinal tissue from human with diabetic retinopathy	Methylation-specific PCR	D-loop region, *MT-COXII* and *MT-CYTB* genes	Increased mtDNA methylation, particularly of the D-loop region, and decreased *CYTB*, *ND6* and *COXII* gene expression levels	[79]
Leukocytes from 32 obese and 8 lean individuals	Methylation-specific PCR	D-loop region	Decreased D-loop methylation and increased mtDNA copy number associated with insulin resistance	[80]
Peripheral blood of individuals that underwent insulin sensitivity, power of fasting glucose (FG) and hemoglobin A1c levels measurements	Methylation-specific PCR	D-loop region and *MT-ND6* gene	D-loop methylation correlated with changes in insulin sensitivity (marker for earlier stage of prediabetes progression) but had marginal interaction with A1c and FG (the later-stage markers in prediabetes progression)	[81]
Buccal swabs from a young Caucasian population (n = 69) with information on body composition (BMI, WHtR and bioimpedance measurements)	Pyrosequencing	D-loop region	D-loop methylation levels were significantly higher in overweight than in lean female subjects, and a specific CpG located in the D-loop associated with impaired body composition. Body composition impairment was predicted by a combined variable including mtDNA copy number and the D-loop methylation (AUC = 0.785; *p* = 0.009).	[82]
Platelet mtDNA from 200 adults with overweight and obesity, of whom 84 developed CVDs	Pyrosequencing	D-loop and light-strand origin of replication (MT-OLR) regions; *MT-CO1, MT-CO2, MT-CO3, MT-TL1* and *MT-TF* genes	Methylation at *MT-CO1*, *MT-CO3* and *MT-TL1* genes was higher in individuals who developed CVDs	[83]
Retinal microvasculature from type 2 (T2D) and type 1 (T1D) diabetes rat models, and high-fat (HF) diet rats	Immunoprecipitation	D-loop region	Increased D-loop methylation in T2D group, compared to T1D or HF groups	[84]
Vessel specimens from patients with carotid occlusive disease, in vitro and in vivo models of arterial stenotic/occlusive diseases	Methylation-specific PCR	D-loop region	DNMT1-mediated D-loop hypermethylation in the intima media layer of mouse and human tissue. The impaired contractility of a ligated vessel could be restored by transplantation of DNMT1-deleted mitochondria.	[85]
Peripheral blood leukocytes from 50 patients with stable coronary artery disease (SCAD) and 50 with acute coronary syndrome (ACS)	Methylation-specific PCR	D-loop region and nuclear *PPARGC1A* gene	Compared to patients with SCAD, those with ACS had significantly lower mtDNA copy number and higher D-loop methylation levels	[86]

**Table 3:** mtDNA methylation studies in senescence and aging.

Experimental Model	Method	mtDNA Region Investigated	Observation	Reference
Mouse brain samples	Enzyme-linked immunosorbent assay and glucosyltransferase assay	D-loop region and *MT-ND2* and *MT-ND5* genes’ 5-hmC content. Global mtDNA content of 5-hmC and 5-mC.	Decreased content of 5-hmC both at a global and a sequence-specific level in frontal cortex, but not in the cerebellum, of mice during aging. Transcript levels of mtDNA genes, including *MT-ND2*, *MT-ND4*, *MT-ND4L* and *MT-ND5*, increased during aging in the frontal cortex.	[52]
Peripheral blood of 381 individuals aged 38–107 years	Bisulfite sequencing	*MT-RNR1* and *MT-RNR2* genes	*MT-RNR1* methylation levels positively associated with increasing age, particularly among women older than 85 years of age. Subjects with higher methylation levels exhibited a mortality risk higher than those with lower levels.	[89]
Replicative and senescent human and mouse endothelial cells	Bisulfite sequencing	D-loop region and *MT-COI* gene	D-loop was demethylated and mtDNA copy number increased in senescent cells with respect to proliferative endothelial cells	[53]
Peripheral blood of 82 individuals aged 18–91 years	Bisulfite sequencing	Methylation levels of 133 CpG sites of mtDNA	Methylation of two CpG sites located within the *MT-RNR1* gene showed an inverse correlation with age	[90]
Senescent mesenchymal stem cells (MSCs) from human fetal heart tissues	Combined bisulfite restriction analysis	Eleven CpG sites located in different mtDNA genes	Three CpG sites were found hypomethylated in senescent cells. One of these CpG sites was located in the *MT-CO1* gene which in turn was up-regulated in MSCs in parallel with the onset of senescence.	[57]
Mouse oocyte maturation, postovulatory oocyte aging and early embryo development	Bisulfite sequencing	D-loop region and *Mt-Rnr1, Mt-rnr2* and ATP genes	Absence of mitochondrial DNA methylation in mouse oocyte maturation, aging and early embryo development	[91]
Two senescence models were constructed, replicative senescence and stress-induced premature senescence, using human heart mesenchymal stem cells (HMSCs)	Bisulfite sequencing	*MT-CO2* gene	Along with the senescence of HMSCs, MT-*CO2* gene methylation increased and its protein expression level significantly decreased. Treatment with 5-aza-2’-deoxycytidine inhibited *COX2* methylation.	[92]

**Table 4:** mtDNA methylation studies in neurodegenerative diseases.

Experimental Model	Method	mtDNA Region Investigated	Observation	Reference
Brain and spinal cord motor neurons of mice, and post-mortem human cortex of 12 ALS patients	Immunohistochemistry	Global 5-mC	Increased DNMT activity and 5-mC mtDNA content in motor neurons	[48]
Superior and middle temporal gyrus (SMTG) and cerebellum (CER) of 7 late-onset AD patients and 5 control subjects	Immunohistochemistry	Global 5-hmC	A trend toward a significant increase in 5-hmC content in SMTG mtDNA of AD patients	[93]
Spinal cord and skeletal muscle of ALS mice and non-transgenic (non-tg) mice	Pyrosequencing	D-loop region and *Mt-rnr2* gene	Higher methylation levels of *Mt-rnr2* gene in spinal cord and skeletal muscle of ALS mice with respect to non-tg mice. Decreased D-loop methylation in spinal cord of mice with G93A *SOD1* mutation with respect to non-tg mice.	[49]
Entorhinal cortex of 8 AD-related pathology patients and 8 control subjects; cerebral cortex of an AD mouse model; substantia nigra of 10 PD patients and 10 control subjects	Pyrosequencing	D-loop region, *MT-ND1* and *MT-ND6* genes	Increased D-loop methylation levels in AD-related pathology patients with respect to control subjects. Dynamic pattern of D-loop methylation in mice along with disease pathology progression. Decreased D-loop methylation in PD patients. *MT-ND1* less methylated in AD-related patients than in control samples.	[94]
Peripheral blood of 133 late-onset AD patients and 130 matched controls	Methylation-sensitive high-resolution melting (MS-HRM)	D-loop region	Significant 25% reduction in D-loop methylation in AD patients	[95]
Peripheral blood of 114 individuals, including 54 ALS patients with mutations in *SOD1*, *TARDBP*, *FUS* or C9orf72 genes, 28 pre-symptomatic carriers of the mutations and 32 noncarrier family members	MS-HRM	D-loop region	*SOD1* mutation carriers showed a significant decrease in D-loop methylation levels. Inverse correlation between D-loop methylation levels and mtDNA copy number.	[96]
Hippocampus from AD model and wild-type mice at 9 months of age	Pyrosequencing	D-loop region and *mt-Rnr1* gene	Decreased D-loop and increased *mt-Rnr1* methylation levels, with concomitant reduction in mtDNA copy number and gene expression in AD mice	[97]
Blood–brain barrier cell line (hCMEC/D3 cells) treated with Aβ1–42 peptide for 12 h and cultured for another 12 h, after withdrawal of Aβ1–42	LC-MS/MS	Global mtDNA methylation	Treatment with Aβ1–42 induced mtDNA hypermethylation. Increased methylation was not restored by removal of Aβ1–42.	[98]
Platelets from 47 patients and 40 healthy control subjects	Pyrosequencing	*MT-TL1*, *MT-CO1*, *MT-CO2* and *MT-CO3* genes	Methylation analysis did not reveal any mtDNA methylation differences between PD patients and control subjects	[99]
Peripheral blood of 63 ALS patients, including 36 sporadic, 14 *SOD1* and 13 *C9orf72* cases, and 51 controls	Pyrosequencing	D-loop region	D-loop methylation levels were significantly lower in ALS patients. Both *SOD1*-related and sporadic ALS patients, but not *C9orf72*-related ones, had lower D-loop methylation levels compared to controls. Significant inverse correlation between D-loop methylation and mtDNA copy number.	[100]
Hippocampus from AD model and wild-type mice	Pyrosequencing	*Cytb* and *Cox2* genes	Hypermethylation of *Cytb* and *Cox2* genes with decreased mtDNA copy numbers and expression in the hippocampi of AD mice	[101]

**Table 5:** mtDNA methylation studies in other diseases.

Experimental Model	Method	mtDNA Region Investigated	Observation	Reference
Lymphoblastoid cells from 6 Down’s syndrome (DS) children and 6 control subjects	Mass spectrometry	Global mtDNA 5-mC content	mtDNA was hypomethylated in DS compared to healthy subjects	[102]
Liver biopsies from 22 patients with non-alcoholic steatohepatitis and 23 patients with simple steatosis	Methylation-specific PCR	D-loop region and *MT-ND6* and *MT-CO1* genes	Higher *MT-ND6* methylation and lower *MT-ND6* gene expression in individuals with simple steatosis. *MT-ND6* methylation status inversely correlated with physical activity.	[103]
Fetal cord blood of newborns from mothers with placenta insufficiency and controls	Pyrosequencing	D-loop region and *MT-RNR1* and *MT-CO1* genes	Decreased D-loop methylation levels in cases compared to controls. D-loop methylation levels were associated with poorer fetal outcomes. Inverse correlation between *MT-CO1* methylation levels and mtDNA content.	[104]
Peripheral blood of 118 patients with major depressive disorder (MDD) and 116 control subjects	Methylation-specific PCR	D-loop region and nuclear *PGC1α* gene promoter	Patients with MDD had a higher mtDNA copy number and decreased DNA methylation in the *PGC1α* gene promoter. D-loop methylation levels did not differ between MDD and control subjects.	[105]
Peripheral blood cells from 70 ADHD subjects and 70 healthy controls	Methylation-specific PCR	D-loop region and nuclear *PPARGC1A* gene	mtDNA copy number was significantly higher in ADHD patients than in controls. Methylation levels of *PPARGC1A* were decreased in ADHD patients compared to controls. D-loop methylation levels did not differ between ADHD and control subjects.	[106]

**Table 6:** Epigenetic effects of exogenous and endogenous molecules and of genetic variants on mtDNA methylation and hydroxymethylation.

Experimental Model	Method	mtDNA region Investigated	Observation	Reference
Effect of valproic acid in mouse 3T3-L1 cells	ELISA and enzymatic restriction digestion	D-loop region and *COX3* and *MT-ND5* genes. Global 5-mC and 5-hmC mtDNA content.	Decreased 5-hmC at global level, in *MT-ND5* and *COX3* genes and in D-loop region	[51]
Peripheral blood of individuals exposed to particulate matter (PM_1_), air benzene and traffic-derived elemental carbon exposure	Pyrosequencing	D-loop region, *MT-TF* and *MT-RNR1* genes	*MT-TF* and *MT-RNR1* methylation levels positively correlated with PM_1_, as well as with mtDNA copy number	[108]
Frontal lobe of Wistar rats exposed to the endocrine disruptor polybrominated dyphenil ether BDE-47	Pyrosequencing, Immunohistochemistry	*Mt-co1*, *Mt-co2* and *Mtco3* mitochondrial genes; *Bdnf* and *Nr3c1* nuclear genes. Global nuclear 5-hmC and 5-mC content.	Decreased methylation levels of *Mt-co2, Bdnf* and *Nr3c1* genes, together with decrease in global DNA methylation in rats exposed to BDE-47	[109]
Effect of PM_2.5_ on human placenta	Pyrosequencing	D-loop region, *MT-RNR1* gene	PM_2.5_ levels positively correlated with both *MT-RNR1* and D-loop methylation and inversely correlated with mtDNA content	[56]
Maternal betaine supplementation during gestation in pregnant sows	5mC Immunoprecipitation followed by qPCR	D-loop region and *mt-Cox1, mt-Cox3* and *mt-Nd4* genes	Decreased D-loop methylation levels and increased expression of mtDNA genes in the muscle of newborn piglets	[110]
Liver of large yellow croakers (*Larimichthys crocea*) fed with different lipid sources (fish oil (FO), palmitic acid, olive oil (OO), sunflower oil or perilla oil (PO))	Pyrosequencing	D-loop region, *MT-TR, MT-ND4L* and *MT-RNR1* genes	*MT-TR* and *MT-NAD4L* methylation higher in the OO and PO than in the FO, whereas *MT-RNR1* methylation was lower in the OO group than in the FO group	[111]
Effect of maternal smoking during pregnancy in placenta and foreskin	Pyrosequencing	D-loop region	Positive association between D-loop methylation and maternal smoking during pregnancy	[112]
Association between exposure to particulate matter (PM_2.5_) and blood mtDNA methylation in relation to heart rate variability (HRV) markers	Pyrosequencing	D-loop region, *MT-TF* and *MT-RNR1* genes	D-loop methylation negatively associated with PM_2.5_ levels. Participants with higher mtDNA methylation levels were more susceptible to the effect of PM_2.5_ on HRV.	[113]
Polycystic ovaries (PCO) of gilts with hyperhomocysteinemia	Bisulfite sequencing	D-loop region and *MT-RNR1, MT-RNR2* and *ND4* genes	mtDNA significantly hypermethylated in all regions analyzed in PCO with respect to healthy ovaries	[114]
Liver of large yellow croaker fed with one of three diets characterized by a low (6%), moderate (12%, the control diet) or high (18%) crude lipid content	Pyrosequencing	D-loop region and *MT-ND6* and *MT-RNR1* genes	D-loop methylation levels higher in the high-lipid than the control group. Increased mtDNA copy number in the high-lipid group.	[115]
Liver of rats fed with high levels of fructose and with normal diet (control group)	Enzyme-linked immunosorbent assay	Global 5-mC and 5-hmC mtDNA content	Global hypomethylation of mtDNA in fructose-fed rats as well as higher mtDNA content and transcription of several mtDNA genes	[116]
Peripheral blood of chrome plating workers and control subjects	Sequenom MassARRAY platform	*MT-TF* and *MT-RNR1* genes	The mtDNA methylation was lower in chrome-exposed compared to control subjects	[117]
Effect of maternal smoking during pregnancy on placenta	Pyrosequencing	D-loop region, and *MT-RNR1* gene; nuclear *CYP1A1* gene	Higher *MT-RNR1* gene methylation levels and lower mtDNA content in placenta samples of smoker pregnant mothers	[118]
Effect of cord blood thyroid hormones thyroxine (T4) and triiodothyronine (FT3) on placenta methylation levels	Pyrosequencing	D-loop region, *MT-RNR1* gene	*MT-RNR1* and D-loop methylation inversely correlated with thyroid hormones levels. Positive association between thyroid hormones and placenta mtDNA content.	[119]
Peripheral blood of welders exposed to respirable dust	Pyrosequencing	D-loop region, *MT-TF* gene	Decreased D-loop and *MT-TF* methylation and increased mtDNA copy number associated with welding fumes exposure	[120]
Hippocampus of rats that received iron in the neonatal period and cannabidiol (CBD) for 14 days in adulthood	Enzyme-linked immunosorbent assay	Global 5-mC and 5-hmC mtDNA content	Iron induced decreased levels of both 5-mC and 5-hmC. CBD induced increase in 5-hmC but not in 5-mC.	[121]
Peripheral blood of individuals exposed to arsenic in drinking water and of control subjects	Methylation-specific PCR	D-loop region and *MT-ND6* gene	D-loop and *MT-ND6* hypomethylation, increased *MT-ND6*, *MT-ND4* and *TFAM* gene expression as well as increased mtDNA copy number in individuals exposed to arsenic	[59]
Glioblastoma multiforme HSR-GBM1 cell line exposed to the DNA demethylation agents 5-azacytidine and vitamin C	MeDIP-Seq	Multiple mtDNA sequences	Reduced mtDNA methylation at most of the regions analyzed	[122]
Association between mtDNA methylation and mitochondrial-derived peptides (MDPs), including HN, MOTS-c and SHLPs in cord blood tissue from newborns exposed to air pollutants during gestation	Pyrosequencing	D-loop, *MT-TF* and *MT-RNR1* genes	Liver tissue from mice exposed for 10 weeks to particulate matter had higher MDP levels. DNA methylation of two distinct regions of the D-Loop was associated with levels of MDPs.	[123]
Frozen platelet pellets from 26 women peripheral blood samples (9 controls, 8 l-leucine and 9 l-leucine + l-carnitine)	Pyrosequencing	D-loop region and *MT-CO1* gene	l-carnitine supplementation increased D-loop methylation in platelets (+6.63%)	[124]
Effect of HIV-1 infection and cocaine, either alone or in combination, on mtDNA methylation in human primary astrocyte	Targeted next-generation bisulfite sequencing, pyrosequencing and ELISA assay	D-loop region, *MT-CYTB*, *MT-RNR1* and *MT-ND1* genes. Global mtDNA methylation.	HIV-1 infection and cocaine exposure reduced global methylation and D-loop, *MT-RNR1*, *MT-ND5*, *MT-ND1* and *MT-CYTB* genes of mtDNA in vitro and altered the expression of DNMTs and TET proteins both in vitro and in vivo	[125]
Porcine oocyte treated with homocysteine	Bisulfite sequencing	*MT-RNR1* and *MT-RNR2* genes	Increased methylation levels of the *MT-RNR2* gene after homocysteine treatment	[126]
Peripheral blood mononuclear cells of 20 healthy individuals were isolated from whole blood and stimulated with lipopolysaccharide (LPS) for 48 h	Restriction enzymes followed by qPCR	D-loop region and *MT-RNR1* gene and *TTF* site	Degree of D-loop methylation slightly decreased. Although only modest alterations were seen in the degree of mtDNA methylation, these strongly correlated with IL-6 and IL-10 expression.	[127]
Correlations among common polymorphisms of genes required for one-carbon metabolism (*MTHFR*, *MTRR*, *MTR* and *RFC-1*) and DNA methylation reactions (*DNMT1*, *DNMT3A* and *DNMT3B*) and mtDNA methylation in peripheral blood of 263 subjects	MS-HRM	D-loop region	*MTRR* 66A > G and *DNMT3A* −448A > G polymorphisms were significantly associated with D-loop methylation levels	[128]
Placental tissue from women who smoked during pregnancy, women with high air pollutant exposure and a control group with low air pollutant exposure	Pyrosequencing	D-loop region; *PINK1*, *DNA2* and *POLG1* nuclear genes	D-loop methylation higher in mothers that smoked extensively or highly exposed to air pollutants. D-loop methylation levels inversely correlated with placental mtDNA content and associated with birth weight.	[129]

## References

[1] Shaughnessy D.T., McAllister K., Worth L., Haugen A.C., Meyer J.N., Domann F.E., Van Houten B., Mostoslavsky R., Bultman S.J., Baccarelli A.A. (2014). Mitochondria; Energetics, epigenetics, and cellular responses to stress. Environ. Health Perspect..

[2] Pittis A.A., Gabaldón T. (2016). Late acquisition of mitochondria by a host with chimaeric prokaryotic ancestry. Nature.

[3] Farge G., Falkenberg M. (2019). Organization of DNA in Mammalian Mitochondria. Int. J. Mol. Sci..

[4] Chocron E.S., Munkácsy E., Pickering A.M. (2019). Cause or casualty: The role of mitochondrial DNA in aging and age-associated disease. Biochim. Biophys. Acta Mol. Basis Dis..

[5] Mposhi A., Van der Wijst M.G., Faber K.N., Rots M.G. (2017). Regulation of mitochondrial gene expression, the epigenetic enigma. Front. Biosci..

[6] Cavalcante G.C., Magalhães L., Ribeiro-Dos-Santos Â., Vidal A.F. (2020). Mitochondrial Epigenetics: Non-Coding RNAs as a Novel Layer of Complexity. Int. J. Mol. Sci..

[7] Manev H., Dzitoyeva S. (2013). Progress in mitochondrial epigenetics. Biomol. Concepts.

[8] Feil R., Fraga M.F. (2012). Epigenetics and the environment: Emerging patterns and implications. Nat. Rev. Genet..

[9] Berdasco M., Esteller M. (2019). Clinical epigenetics: Seizing opportunities for translation. Nat. Rev. Genet..

[10] Coppedè F., Stoccoro A. (2019). Mitoepigenetics and Neurodegenerative Diseases. Front. Endocrinol..

[11] Bannister A.J., Kouzarides T. (2011). Regulation of chromatin by histone modifications. Cell Res..

[12] Jones P.A. (2012). Functions of DNA methylation: Islands, start sites, gene bodies and beyond. Nat. Rev. Genet..

[13] Andrey G., Mundlos S. (2017). The three-dimensional genome: Regulating gene expression during pluripotency and development. Development.

[14] Moore L.D., Le T., Fan G. (2013). DNA methylation and its basic function. Neuropsychopharmacology.

[15] Coppedè F. (2020). One-carbon epigenetics and redox biology of neurodegeneration. Free Radic. Biol. Med..

[16] Christopher M.A., Kyle S.M., Katz D.J. (2017). Neuroepigenetic mechanisms in disease. Epigenet. Chromatin.

[17] Zampieri M., Ciccarone F., Calabrese R., Franceschi C., Bürkle A., Caiafa P. (2015). Reconfiguration of DNA methylation in aging. Mech. Ageing Dev..

[18] Jang H.S., Shin W.J., Lee J.E., Do J.T. (2017). CpG and Non-CpG Methylation in Epigenetic Gene Regulation and Brain Function. Genes.

[19] Richa R., Sinha R.P. (2014). Hydroxymethylation of DNA: An epigenetic marker. EXCLI J..

[20] Barnes C.E., English D.M., Cowley S.M. (2019). Acetylation & Co: An expanding repertoire of histone acylations regulates chromatin and transcription. Essays Biochem..

[21] Peschansky V.J., Wahlestedt C. (2014). Non-coding RNAs as direct and indirect modulators of epigenetic regulation. Epigenetics.

[22] Wilczynska A., Bushell M. (2015). The complexity of miRNA-mediated repression. Cell Death Differ..

[23] Ghosh S., Singh K.K., Sengupta S., Scaria V. (2015). Mitoepigenetics: The different shades of grey. Mitochondrion.

[24] De Paepe B. (2019). How mitochondrial DNA-driven changes to chromosomal DNA methylation add a layer of complexity to mitochondrial disease. Epigenomics.

[25] Wiese M., Bannister A.J. (2020). Two genomes, one cell: Mitochondrial-nuclear coordination via epigenetic pathways. Mol. Metab..

[26] Bacalini M.G., D’Aquila P., Marasco E., Nardini C., Montesanto A., Franceschi C., Passarino G., Garagnani P., Bellizzi D. (2017). The methylation of nuclear and mitochondrial DNA in ageing phenotypes and longevity. Mech. Ageing Dev..

[27] Smiraglia D.J., Kulawiec M., Bistulfi G.L., Gupta S.G., Singh K.K. (2008). A novel role for mitochondria in regulating epigenetic modification in the nucleus. Cancer Biol. Ther..

[28] Bellizzi D., D’Aquila P., Giordano M., Montesanto A., Passarino G. (2012). Global DNA methylation levels are modulated by mitochondrial DNA variants. Epigenomics.

[29] Vivian C.J., Brinker A.E., Graw S., Koestler D.C., Legendre C., Gooden G.C., Salhia B., Welch D.R. (2017). Mitochondrial Genomic Backgrounds Affect Nuclear DNA Methylation and Gene Expression. Cancer Res..

[30] Castellani C.A., Longchamps R.J., Sumpter J.A., Newcomb C.E., Lane J.A., Grove M.L., Bressler J., Brody J.A., Floyd J.S., Bartz T.M. (2020). Mitochondrial DNA copy number can influence mortality and cardiovascular disease via methylation of nuclear DNA CpGs. Genome Med..

[31] Kelly R.D., Mahmud A., McKenzie M., Trounce I.A., St John J.C. (2012). Mitochondrial DNA copy number is regulated in a tissue specific manner by DNA methylation of the nuclear-encoded DNA polymerase gamma A. Nucleic Acids Res..

[32] Lee W., Johnson J., Gough D.J., Donoghue J., Cagnone G.L., Vaghjiani V., Brown K.A., Johns T.G., St John J.C. (2015). Mitochondrial DNA copy number is regulated by DNA methylation and demethylation of POLGA in stem and cancer cells and their differentiated progeny. Cell Death Dis..

[33] Chen K., Lu P., Beeraka N.M., Sukocheva O.A., Madhunapantula S.V., Liu J., Sinelnikov M.Y., Nikolenko V.N., Bulygin K.V., Mikhaleva L.M. (2020). Mitochondrial mutations and mitoepigenetics: Focus on regulation of oxidative stress-induced responses in breast cancers. Semin. Cancer Biol..

[34] Hong E.E., Okitsu C.Y., Smith A.D., Hsieh C.L. (2013). Regionally specific and genome-wide analyses conclusively demonstrate the absence of CpG methylation in human mitochondrial DNA. Mol. Cell. Biol..

[35] Mechta M., Ingerslev L.R., Fabre O., Picard M., Barrès R. (2017). Evidence Suggesting Absence of Mitochondrial DNA Methylation. Front. Genet..

[36] Matsuda S., Yasukawa T., Sakaguchi Y., Ichiyanagi K., Unoki M., Gotoh K., Fukuda K., Sasaki H., Suzuki T., Kang D. (2018). Accurate estimation of 5-methylcytosine in mammalian mitochondrial DNA. Sci. Rep..

[37] Vanyushin B.F., Kiryanov G.I., Kudryashova I.B., Belozersky A.N. (1971). DNA-methylase in loach embryos (Misgurnus fossilis). FEBS Lett..

[38] Nass M.M. (1973). Differential methylation of mitochondrial and nuclear DNA in cultured mouse, hamster and virus-transformed hamster cells. In vivo and in vitro methylation. J. Mol. Biol..

[39] Cummings D.J., Tait A., Goddard J.M. (1974). Methylated bases in DNA from Paramecium aurelia. Biochim. Biophys. Acta.

[40] Shmookler Reis R.J., Goldstein S. (1983). Mitochondrial DNA in mortal and immortal human cells. Genome number, integrity, and methylation. J. Biol. Chem..

[41] Pollack Y., Kasir J., Shemer R., Metzger S., Szyf M. (1984). Methylation pattern of mouse mitochondrial DNA. Nucleic Acids Res..

[42] Maekawa M., Taniguchi T., Higashi H., Sugimura H., Sugano K., Kanno T. (2004). Methylation of mitochondrial DNA is not a useful marker for cancer detection. Clin. Chem..

[43] Rebelo A.P., Williams S.L., Moraes C.T. (2009). In vivo methylation of mtDNA reveals the dynamics of protein-mtDNA interactions. Nucleic Acids Res..

[44] Shock L.S., Thakkar P.V., Peterson E.J., Moran R.G., Taylor S.M. (2011). DNA methyltransferase 1, cytosine methylation, and cytosine hydroxymethylation in mammalian mitochondria. Proc. Natl. Acad. Sci. USA.

[45] Dostal V., Churchill M.E.A. (2019). Cytosine methylation of mitochondrial DNA at CpG sequences impacts transcription factor A DNA binding and transcription. Biochim. Biophys. Acta Gene Regul. Mech..

[46] Bellizzi D., D’Aquila P., Scafone T., Giordano M., Riso V., Riccio A., Passarino G. (2013). The control region of mitochondrial DNA shows an unusual CpG and non-CpG methylation pattern. DNA Res..

[47] Saini S.K., Mangalhara K.C., Prakasam G., Bamezai R.N.K. (2017). DNA Methyltransferase1 (DNMT1) Isoform3 methylates mitochondrial genome and modulates its biology. Sci. Rep..

[48] Chestnut B.A., Chang Q., Price A., Lesuisse C., Wong M., Martin L.J. (2011). Epigenetic regulation of motor neuron cell death through DNA methylation. J. Neurosci..

[49] Wong M., Gertz B., Chestnut B.A., Martin L.J. (2013). Mitochondrial DNMT3A and DNA methylation in skeletal muscle and CNS of transgenic mouse models of ALS. Front. Cell. Neurosci..

[50] Dou X., Boyd-Kirkup J.D., McDermott J., Zhang X., Li F., Rong B., Zhang R., Miao B., Chen P., Cheng H. (2019). The strand-biased mitochondrial DNA methylome and its regulation by DNMT3A. Genome Res..

[51] Chen H., Dzitoyeva S., Manev H. (2012). Effect of valproic acid on mitochondrial epigenetics. Eur. J. Pharmacol..

[52] Dzitoyeva S., Chen H., Manev H. (2012). Effect of aging on 5-hydroxymethylcytosine in brain mitochondria. Neurobiol. Aging.

[53] Bianchessi V., Vinci M.C., Nigro P., Rizzi V., Farina F., Capogrossi M.C., Pompilio G., Gualdi V., Lauri A. (2016). Methylation profiling by bisulfite sequencing analysis of the mtDNA Non-Coding Region in replicative and senescent Endothelial Cells. Mitochondrion.

[54] van der Wijst M.G., van Tilburg A.Y., Ruiters M.H., Rots M.G. (2017). Experimental mitochondria-targeted DNA methylation identifies GpC methylation, not CpG methylation, as potential regulator of mitochondrial gene expression. Sci. Rep..

[55] Gao J., Wen S., Zhou H., Feng S. (2015). De-methylation of displacement loop of mitochondrial DNA is associated with increased mitochondrial copy number and nicotinamide adenine dinucleotide subunit 2 expression in colorectal cancer. Mol. Med. Rep..

[56] Janssen B.G., Byun H.M., Gyselaers W., Lefebvre W., Baccarelli A.A., Nawrot T.S. (2015). Placental mitochondrial methylation and exposure to airborne particulate matter in the early life environment: An ENVIRONAGE birth cohort study. Epigenetics.

[57] Yu D., Du Z., Pian L., Li T., Wen X., Li W., Kim S.J., Xiao J., Cohen P., Cui J. (2017). Mitochondrial DNA Hypomethylation Is a Biomarker Associated with Induced Senescence in Human Fetal Heart Mesenchymal Stem Cells. Stem Cells Int..

[58] Feng S., Xiong L., Ji Z., Cheng W., Yang H. (2012). Correlation between increased ND2 expression and demethylated displacement loop of mtDNA in colorectal cancer. Mol. Med. Rep..

[59] Sanyal T., Bhattacharjee P., Bhattacharjee S., Bhattacharjee P. (2018). Hypomethylation of mitochondrial D-loop and ND6 with increased mitochondrial DNA copy number in the arsenic-exposed population. Toxicology.

[60] Sun Z., Terragni J., Borgaro J.G., Liu Y., Yu L., Guan S., Wang H., Sun D., Cheng X., Zhu Z. (2013). High-resolution enzymatic mapping of genomic 5-hydroxymethylcytosine in mouse embryonic stem cells. Cell Rep..

[61] Patil V., Cuenin C., Chung F., Aguilera J.R.R., Fernandez-Jimenez N., Romero-Garmendia I., Bilbao J.R., Cahais V., Rothwell J., Herceg Z. (2019). Human mitochondrial DNA is extensively methylated in a non-CpG context. Nucleic Acids Res..

[62] de Lima C.B., Sirard M.A. (2020). Mitoepigenetics: Methylation of mitochondrial DNA is strand-biased in bovine oocytes and embryos. Reprod. Domest. Anim..

[63] Ghosh S., Sengupta S., Scaria V. (2016). Hydroxymethyl cytosine marks in the human mitochondrial genome are dynamic in nature. Mitochondrion.

[64] Xiao C.L., Zhu S., He M., Chen D., Zhang Q., Chen Y., Yu G., Liu J., Xie S.Q., Luo F. (2018). N6-Methyladenine DNA Modification in the Human Genome. Mol. Cell..

[65] Koh C.W.Q., Goh Y.T., Toh J.D.W., Neo S.P., Ng S.B., Gunaratne J., Gao Y.G., Quake S.R., Burkholder W.F., Goh W.S.S. (2018). Single-nucleotide-resolution sequencing of human N6-methyldeoxyadenosine reveals strand-asymmetric clusters associated with SSBP1 on the mitochondrial genome. Nucleic Acids Res..

[66] Hao Z., Wu T., Cui X., Zhu P., Tan C., Dou X., Hsu K.W., Lin Y.T., Peng P.H., Zhang L.S. (2020). N6-Deoxyadenosine Methylation in Mammalian Mitochondrial DNA. Mol. Cell.

[67] Dong Z., Pu L., Cui H. (2020). Mitoepigenetics and Its Emerging Roles in Cancer. Front. Cell Dev. Biol..

[68] Devall M., Roubroeks J., Mill J., Weedon M., Lunnon K. (2016). Epigenetic regulation of mitochondrial function in neurodegenerative disease: New insights from advances in genomic technologies. Neurosci. Lett..

[69] Sun C., Reimers L.L., Burk R.D. (2011). Methylation of HPV16 genome CpG sites is associated with cervix precancer and cancer. Gynecol. Oncol..

[70] Tong H., Zhang L., Gao J., Wen S., Zhou H., Feng S. (2017). Methylation of mitochondrial DNA displacement loop region regulates mitochondrial copy number in colorectal cancer. Mol. Med. Rep..

[71] Han X., Zhao Z., Zhang M., Li G., Yang C., Du F., Wang J., Zhang Y., Wang Y., Jia Y. (2017). Maternal trans-general analysis of the human mitochondrial DNA pattern. Biochem. Biophys. Res. Commun..

[72] Morris M.J., Hesson L.B., Poulos R.C., Ward R.L., Wong J.W.H., Youngson N.A. (2018). Reduced nuclear DNA methylation and mitochondrial transcript changes in adenomas do not associate with mtDNA methylation. Biomark. Res..

[73] Sun X., Vaghjiani V., Jayasekara W.S.N., Cain J.E., St John J.C. (2018). The degree of mitochondrial DNA methylation in tumor models of glioblastoma and osteosarcoma. Clin. Epigenet..

[74] Aminuddin A., Ng P.Y., Leong C.O., Chua E.W. (2020). Mitochondrial DNA alterations may influence the cisplatin responsiveness of oral squamous cell carcinoma. Sci. Rep..

[75] Goldsmith C., Rodríguez-Aguilera J.R., El-Rifai I., Jarretier-Yuste A., Hervieu V., Raineteau O., Saintigny S., de Sánchez V.C., Dante R., Ichim G. (2021). Low biological fluctuation of mitochondrial CpG and non-CpG methylation at the single-molecule level. Sci. Rep..

[76] Perrone-Filardi P., Paolillo S., Costanzo P., Savarese G., Trimarco B., Bonow R.O. (2015). The role of metabolic syndrome in heart failure. Eur. Heart J..

[77] Ren J., Pulakat L., Whaley-Connell A., Sowers J.R. (2010). Mitochondrial biogenesis in the metabolic syndrome and cardiovascular disease. J. Mol. Med..

[78] Baccarelli A.A., Byun H.M. (2015). Platelet mitochondrial DNA methylation: A potential new marker of cardiovascular disease. Clin. Epigenetics.

[79] Mishra M., Kowluru R.A. (2015). Epigenetic Modification of Mitochondrial DNA in the Development of Diabetic Retinopathy. Investig. Ophthalmol. Vis. Sci..

[80] Zheng L.D., Linarelli L.E., Liu L., Wall S.S., Greenawald M.H., Seidel R.W., Estabrooks P.A., Almeida F.A., Cheng Z. (2015). Insulin resistance is associated with epigenetic and genetic regulation of mitochondrial DNA in obese humans. Clin. Epigenetics.

[81] Zheng L.D., Linarelli L.E., Brooke J., Smith C., Wall S.S., Greenawald M.H., Seidel R.W., Estabrooks P.A., Almeida F.A., Cheng Z. (2016). Mitochondrial Epigenetic Changes Link to Increased Diabetes Risk and Early-Stage Prediabetes Indicator. Oxid. Med. Cell. Longev..

[82] Bordoni L., Smerilli V., Nasuti C., Gabbianelli R. (2019). Mitochondrial DNA methylation and copy number predict body composition in a young female population. J. Transl. Med..

[83] Corsi S., Iodice S., Vigna L., Cayir A., Mathers J.C., Bollati V., Byun H.M. (2020). Platelet mitochondrial DNA methylation predicts future cardiovascular outcome in adults with overweight and obesity. Clin. Epigenetics.

[84] Kowluru R.A. (2020). Retinopathy in a Diet-Induced Type 2 Diabetic Rat Model and Role of Epigenetic Modifications. Diabetes.

[85] Liu Y.F., Zhu J.J., Yu Tian X., Liu H., Zhang T., Zhang Y.P., Xie S.A., Zheng M., Kong W., Yao W.J. (2020). Hypermethylation of mitochondrial DNA in vascular smooth muscle cells impairs cell contractility. Cell Death Dis..

[86] Park S.H., Lee S.Y., Kim S.A. (2021). Mitochondrial DNA Methylation Is Higher in Acute Coronary Syndrome Than in Stable Coronary Artery Disease. In Vivo.

[87] Childs B.G., Durik M., Baker D.J., van Deursen J.M. (2015). Cellular senescence in aging and age-related disease: From mechanisms to therapy. Nat. Med..

[88] Sun N., Youle R.J., Finkel T. (2016). The Mitochondrial Basis of Aging. Mol. Cell..

[89] D’Aquila P., Giordano M., Montesanto A., De Rango F., Passarino G., Bellizzi D. (2015). Age-and gender-related pattern of methylation in the MT-RNR1 gene. Epigenomics.

[90] Mawlood S.K., Dennany L., Watson N., Dempster J., Pickard B.S. (2016). Quantification of global mitochondrial DNA methylation levels and inverse correlation with age at two CpG sites. Aging.

[91] Fan L.H., Wang Z.B., Li Q.N., Meng T.G., Dong M.Z., Hou Y., Ouyang Y.C., Schatten H., Sun Q.Y. (2019). Absence of mitochondrial DNA methylation in mouse oocyte maturation, aging and early embryo development. Biochem. Biophys. Res. Commun..

[92] Sun X., Wang Z., Cong X., Lv Y., Li Z., Rong L., Yang T., Yu D. (2021). Mitochondrial gene COX2 methylation and downregulation is a biomarker of aging in heart mesenchymal stem cells. Int. J. Mol. Med..

[93] Bradley-Whitman M.A., Lovell M.A. (2013). Epigenetic changes in the progression of Alzheimer’s disease. Mech. Ageing Dev..

[94] Blanch M., Mosquera J.L., Ansoleaga B., Ferrer I., Barrachina M. (2016). Altered Mitochondrial DNA Methylation Pattern in Alzheimer Disease-Related Pathology and in Parkinson Disease. Am. J. Pathol..

[95] Stoccoro A., Siciliano G., Migliore L., Coppedè F. (2017). Decreased Methylation of the Mitochondrial D-Loop Region in Late-Onset Alzheimer’s Disease. J. Alzheimer’s Dis..

[96] Stoccoro A., Mosca L., Carnicelli V., Cavallari U., Lunetta C., Marocchi A., Migliore L., Coppedè F. (2018). Mitochondrial DNA copy number and D-loop region methylation in carriers of amyotrophic lateral sclerosis gene mutations. Epigenomics.

[97] Xu Y., Xu L., Han M., Liu X., Li F., Zhou X., Wang Y., Bi J. (2019). Altered mitochondrial DNA methylation and mitochondrial DNA copy number in an APP/PS1 transgenic mouse model of Alzheimer disease. Biochem. Biophys. Res. Commun..

[98] Liu H., Zhang H., Zhang Y., Xu S., Zhao H., He H., Liu X. (2020). Modeling mtDNA hypermethylation vicious circle mediating Aβ-induced endothelial damage memory in HCMEC/D3 cell. Aging.

[99] Sharma A., Schaefer S.T., Sae-Lee C., Byun H.M., Wüllner U. (2021). Elevated serum mitochondrial DNA in females and lack of altered platelet mitochondrial methylation in patients with Parkinson’s disease. Int. J. Neurosci..

[100] Stoccoro A., Smith A.R., Mosca L., Marocchi A., Gerardi F., Lunetta C., Cereda C., Gagliardi S., Lunnon K., Migliore L. (2020). Reduced mitochondrial D-loop methylation levels in sporadic amyotrophic lateral sclerosis. Clin. Epigenet..

[101] Xu Y., Cheng L., Sun J., Li F., Liu X., Wei Y., Han M., Zhu Z., Bi J., Lai C. (2021). Hypermethylation of Mitochondrial Cytochrome b and Cytochrome c Oxidase II Genes with Decreased Mitochondrial DNA Copy Numbers in the APP/PS1 Transgenic Mouse Model of Alzheimer’s Disease. Neurochem. Res..

[102] Infantino V., Castegna A., Iacobazzi F., Spera I., Scala I., Andria G., Iacobazzi V. (2011). Impairment of methyl cycle affects mitochondrial methyl availability and glutathione level in Down’s syndrome. Mol. Genet. Metab..

[103] Pirola C.J., Gianotti T.F., Burgueño A.L., Rey-Funes M., Loidl C.F., Mallardi P., Martino J.S., Castaño G.O., Sookoian S. (2013). Epigenetic modification of liver mitochondrial DNA is associated with histological severity of nonalcoholic fatty liver disease. Gut.

[104] Novielli C., Mandò C., Tabano S., Anelli G.M., Fontana L., Antonazzo P., Miozzo M., Cetin I. (2017). Mitochondrial DNA content and methylation in fetal cord blood of pregnancies with placental insufficiency. Placenta.

[105] Chung J.K., Lee S.Y., Park M., Joo E.J., Kim S.A. (2019). Investigation of mitochondrial DNA copy number in patients with major depressive disorder. Psychiatry Res..

[106] Kim J.I., Lee S.Y., Park M., Kim S.Y., Kim J.W., Kim S.A., Kim B.N. (2019). Peripheral Mitochondrial DNA Copy Number is Increased in Korean Attention-Deficit Hyperactivity Disorder Patients. Front. Psychiatry.

[107] Alegría-Torres J.A., Baccarelli A., Bollati V. (2011). Epigenetics and lifestyle. Epigenomics.

[108] Byun H.M., Panni T., Motta V., Hou L., Nordio F., Apostoli P., Bertazzi P.A., Baccarelli A.A. (2013). Effects of airborne pollutants on mitochondrial DNA methylation. Part. Fibre Toxicol..

[109] Byun H.M., Benachour N., Zalko D., Frisardi M.C., Colicino E., Takser L., Baccarelli A.A. (2015). Epigenetic effects of low perinatal doses of flame retardant BDE-47 on mitochondrial and nuclear genes in rat offspring. Toxicology.

[110] Jia Y., Song H., Gao G., Cai D., Yang X., Zhao R. (2015). Maternal Betaine Supplementation during Gestation Enhances Expression of mtDNA-Encoded Genes through D-Loop DNA Hypomethylation in the Skeletal Muscle of Newborn Piglets. J. Agric. Food Chem..

[111] Liao K., Yan J., Mai K., Ai Q. (2015). Dietary Olive and Perilla Oils Affect Liver Mitochondrial DNA Methylation in Large Yellow Croakers. J. Nutr..

[112] Armstrong D.A., Green B.B., Blair B.A., Guerin D.J., Litzky J.F., Chavan N.R., Pearson K.J., Marsit C.J. (2016). Maternal smoking during pregnancy is associated with mitochondrial DNA methylation. Environ. Epigenet..

[113] Byun H.M., Colicino E., Trevisi L., Fan T., Christiani D.C., Baccarelli A.A. (2016). Effects of Air Pollution and Blood Mitochondrial DNA Methylation on Markers of Heart Rate Variability. J. Am. Heart Assoc..

[114] Jia L., Li J., He B., Jia Y., Niu Y., Wang C., Zhao R. (2016). Abnormally activated one-carbon metabolic pathway is associated with mtDNA hypermethylation and mitochondrial malfunction in the oocytes of polycystic gilt ovaries. Sci. Rep..

[115] Liao K., Yan J., Mai K., Ai Q. (2016). Dietary lipid concentration affects liver mitochondrial DNA copy number; gene expression and DNA methylation in large yellow croaker (*Larimichthys crocea*). Comp. Biochem. Physiol. B Biochem. Mol. Biol..

[116] Yamazaki M., Munetsuna E., Yamada H., Ando Y., Mizuno G., Murase Y., Kondo K., Ishikawa H., Teradaira R., Suzuki K. (2016). Fructose consumption induces hypomethylation of hepatic mitochondrial DNA in rats. Life Sci..

[117] Yang L., Xia B., Yang X., Ding H., Wu D., Zhang H., Jiang G., Liu J., Zhuang Z. (2016). Mitochondrial DNA hypomethylation in chrome plating workers. Toxicol. Lett..

[118] Janssen B.G., Gyselaers W., Byun H.M., Roles H.A., Cuypers A., Baccarelli A.A., Nawrot T.S. (2017). Placental mitochondrial DNA and CYP1A1 gene methylation as molecular signatures for tobacco smoke exposure in pregnant women and the relevance for birth weight. J. Transl. Med..

[119] Janssen B.G., Byun H.M., Roles H.A., Gyselaers W., Penders J., Baccarelli A.A., Nawrot T.S. (2017). Regulating role of fetal thyroid hormones on placental mitochondrial DNA methylation: Epidemiological evidence from the ENVIRONAGE birth cohort study. Clin. Epigenet..

[120] Xu Y., Li H., Hedmer M., Hossain M.B., Tinnerberg H., Broberg K., Albin M. (2017). Occupational exposure to particles and mitochondrial DNA—Relevance for blood pressure. Environ. Health.

[121] da Silva V.K., de Freitas B.S., Dornelles V.C., Kist L.W., Bogo M.R., Silva M.C., Streck E.L., Hallak J.E., Zuardi A.W., Crippa J.A.S. (2018). Novel insights into mitochondrial molecular targets of iron-induced neurodegeneration: Reversal by cannabidiol. Brain Res. Bull..

[122] Sun X., Johnson J., St John J.C. (2018). Global DNA methylation synergistically regulates the nuclear and mitochondrial genomes in glioblastoma cells. Nucleic Acids Res..

[123] Breton C.V., Song A.Y., Xiao J., Kim S.J., Mehta H.H., Wan J., Yen K., Sioutas C., Lurmann F., Xue S. (2019). Effects of air pollution on mitochondrial function, mitochondrial DNA methylation, and mitochondrial peptide expression. Mitochondrion.

[124] Bordoni L., Sawicka A.K., Szarmach A., Winklewski P.J., Olek R.A., Gabbianelli R. (2020). A Pilot Study on the Effects of l-Carnitine and Trimethylamine-N-Oxide on Platelet Mitochondrial DNA Methylation and CVD Biomarkers in Aged Women. Int. J. Mol. Sci..

[125] Doke M., Jeganathan V., McLaughlin J.P., Samikkannu T. (2020). HIV-1 Tat and cocaine impact mitochondrial epigenetics: Effects on DNA methylation. Epigenetics.

[126] Jia L., Zeng Y., Hu Y., Liu J., Yin C., Niu Y., Wang C., Li J., Jia Y., Hong J. (2019). Homocysteine impairs porcine oocyte quality via deregulation of one-carbon metabolism and hypermethylation of mitochondrial DNA. Biol. Reprod..

[127] Koos B., Moderegger E.L., Rump K., Nowak H., Willemsen K., Holtkamp C., Thon P., Adamzik M., Rahmel T. (2020). LPS-Induced Endotoxemia Evokes Epigenetic Alterations in Mitochondrial DNA That Impacts Inflammatory Response. Cells.

[128] Stoccoro A., Tannorella P., Migliore L., Coppedè F. (2020). Polymorphisms of genes required for methionine synthesis and DNA methylation influence mitochondrial DNA methylation. Epigenomics.

[129] Vos S., Nawrot T.S., Martens D.S., Byun H.M., Janssen B.G. (2021). Mitochondrial DNA methylation in placental tissue: A proof of concept study by means of prenatal environmental stressors. Epigenetics.

[130] Kishita Y., Pajak A., Bolar N.A., Marobbio C.M., Maffezzini C., Miniero D.V., Monné M., Kohda M., Stranneheim H., Murayama K. (2015). Intra-mitochondrial Methylation Deficiency Due to Mutations in SLC25A26. Am. J. Hum. Genet..

[131] Maresca A., Zaffagnini M., Caporali L., Carelli V., Zanna C. (2015). DNA methyltransferase 1 mutations and mitochondrial pathology: Is mtDNA methylated?. Front. Genet..

[132] Liu B., Du Q., Chen L., Fu G., Li S., Fu L., Zhang X., Ma C., Bin C. (2016). CpG methylation patterns of human mitochondrial DNA. Sci. Rep..

[133] Dawid I.B. (1974). 5-methylcytidylic acid: Absence from mitochondrial DNA of frogs and HeLa cells. Science.

[134] Groot G.S., Kroon A.M. (1979). Mitochondrial DNA from various organisms does not contain internally methylated cytosine in -CCGG- sequences. Biochim. Biophys. Acta..

[135] Owa C., Poulin M., Yan L., Shioda T. (2018). Technical adequacy of bisulfite sequencing and pyrosequencing for detection of mitochondrial DNA methylation: Sources and avoidance of false-positive detection. PLoS ONE.

[136] Pawar T., Eide L. (2017). Pitfalls in mitochondrial epigenetics. Mitochondrial DNA Part A.

[137] Devall M., Smith R.G., Jeffries A., Hannon E., Davies M.N., Schalkwyk L., Mill J., Weedon M., Lunnon K. (2017). Regional differences in mitochondrial DNA methylation in human post-mortem brain tissue. Clin. Epigenetics.

[138] Cedar H., Bergman Y. (2009). Linking DNA methylation and histone modification: Patterns and paradigms. Nat. Rev. Genet..

